# Modeling Access Across the Digital Divide for Intersectional Groups Seeking Web-Based Health Information: National Survey

**DOI:** 10.2196/32678

**Published:** 2022-03-15

**Authors:** Kristina Medero, Kelly Merrill Jr, Morgan Quinn Ross

**Affiliations:** 1 School of Communication Ohio State University Columbus, OH United States

**Keywords:** Black, African American, first-level digital divide, health disparities, home computer, internet access, intersectionality, Latino, Latine, Hispanic, mobile, online health information seeking, public computer, structural equation modeling, work computer, mobile phone

## Abstract

**Background:**

The digital divide refers to technological disparities based on demographic characteristics (eg, race and ethnicity). Lack of physical access to the internet inhibits online health information seeking (OHIS) and exacerbates health disparities. Research on the digital divide examines where and how people access the internet, whereas research on OHIS investigates how intersectional identities influence OHIS. We combine these perspectives to explicate how unique context–device access pairings operate differently across intersectional identities—particularly racial and ethnic groups—in the domain of OHIS.

**Objective:**

This study aims to examine how different types of internet access relate to OHIS for different racial and ethnic groups. We investigate relationships among predisposing characteristics (ie, age, sex, education, and income), internet access (home computer, public computer, work computer, and mobile), health needs, and OHIS.

**Methods:**

Analysis was conducted using data from the 2019 Health Information National Trends Survey. Our theoretical model of OHIS explicates the roles of internet access and health needs for racial and ethnic minority groups’ OHIS. Participant responses were analyzed using structural equation modeling. Three separate group structural equation modeling models were specified based on Black, Latine, and White self-categorizations.

**Results:**

Overall, predisposing characteristics (ie, age, sex, education, and income) were associated with internet access, health needs, and OHIS; internet access was associated with OHIS; and health needs were associated with OHIS. Home computer and mobile access were most consistently associated with OHIS. Several notable linkages between predisposing characteristics and internet access differed for Black and Latine individuals. Older racial and ethnic minorities tended to access the internet on home and public computers less frequently; home computer access was a stronger predictor of OHIS for White individuals, and mobile access was a stronger predictor of OHIS for non-White individuals.

**Conclusions:**

Our findings necessitate a deeper unpacking of how physical internet access, the foundational and multifaceted level of the digital divide, affects specific racial and ethnic groups and their OHIS. We not only find support for prior work on the digital divide but also surface new insights, including distinct impacts of context–device access pairings for OHIS and several relationships that differ between racial and ethnic groups. As such, we propose interventions with an intersectional approach to access to ameliorate the impact of the digital divide.

## Introduction

### Background

The benefits of eHealth, or the use of the internet to facilitate health behaviors (eg, online health information seeking [OHIS]) [1], are counteracted by the digital divide. The digital divide was first used to emphasize that racial and ethnic minorities and individuals of lower socioeconomic status did not adopt new technologies to the same extent as White individuals or those of higher socioeconomic status [2]. Obtaining physical access to new technologies and, thus, web-based health information remains a paramount obstacle, particularly for Black or African American (hereafter *Black*) and Latino or Latine or Hispanic (hereafter *Latine*) individuals [3]. This is problematic, as racial and ethnic minorities are more likely to live in areas of concentrated poverty that coincide with limited health care access [4-6]. Systematic inequalities in internet access and health care for racial and ethnic minorities reinforce one another, such that those who would potentially benefit the most from OHIS are often unable to access it.

However, internet access (hereafter *access*) is not monolithic and comprises the use of different devices (eg, smartphone or computer) in various contexts (eg, at home or in public) [7]. Although mobile devices are increasingly more accessible than computers, they can be harder to navigate because of their smaller interfaces [8]. Publicly accessible devices may extend access to those who do not own such devices; however, they often entail irregular availability, which can compound poor health outcomes for minority groups [9]. Recognizing the multidimensionality of access [7] is key to understanding how access via a myriad of devices in various contexts differentially influences OHIS. Furthermore, positioning the digital divide as a health disparity is imperative to developing effective interventions [10]. As such, this study uses data from the 2019 Health Information National Trends Survey (HINTS), also known as HINTS 5, Cycle 3 [11], to bolster theoretical models of OHIS with a nuanced conceptualization of access. We also advance the perspective that the digital divide is a health disparity by applying an intersectional focus to examine how relationships with access and OHIS differ across racial and ethnic groups.

### Theoretical Framework: OHIS Model

The internet has become one of the most common ways of accessing health information [12]. Health information seeking refers to those actions that individuals use to search for information about their health, risks, illnesses, and health-protective behaviors [13]. When conducted on the web (ie, OHIS), seeking out health information can positively affect health outcomes by improving the quality, expense, and efficiency of health care [10]. In addition, OHIS has demonstrated that individuals are more willing to comply with their health decisions [14]. However, those with limited access to OHIS may not experience its benefits. Health disparities faced by low-income and minority communities may be magnified by the digital divide [3,15]. However, when underserved communities are provided the means to participate in OHIS, they gain more health knowledge [16]. Thus, understanding how the digital divide affects OHIS is imperative to enhance the impact of interventions aimed at increasing access among these communities.

The digital divide first highlighted that certain groups of people (eg, racial and ethnic minorities and individuals of low socioeconomic status) lagged in adopting new technologies. This gradual diffusion represents the first-level factor of the digital divide, which has been situated in issues related to ownership, availability, and affordability of the technology [17]. Recent studies have identified additional second-level factors that may also impede technological adoption (eg, skills) [15,18]. Although the focus of OHIS has shifted away from access as some suggest that it has become democratized [19], we argue that it has not been democratized across devices and contexts of use as the lack of physical access remains an obstacle for marginalized groups [3,20]. Moreover, access is heterogeneous, as people can access the internet on multiple devices and at various places [7,21]. Even in populations with saturated home access, disparities can persist for other points of access and the cost to maintain them [7]. Thus, a nuanced conceptualization of access can respond to criticism that the digital divide suggests a simple binary between those who have access and those who do not [22].

Notably, some scholars have applied this multifaceted conceptualization of access to predict the likelihood of web-based activities (including OHIS). Hassani [23] found that people engaged in more OHIS as they increased their points of access (eg, home and work vs only home). Similarly, Mossberger et al [24] found that home computer access is vital to reap the benefits of web-based activities such as OHIS. Although the authors highlight the potential for mobile devices to attenuate the impacts of the digital divide, mobile access alone did little to minimize these impacts in low-income areas. Reisdorf et al [21] also suggest that simply increasing access does not lead to equal results across different contexts. However, these studies investigated OHIS as one of many web-based activities; as such, they were not grounded in theoretical models of OHIS. Moreover, studies that focused on OHIS [25,26] did not examine the impact of specific devices or contexts of use; instead, they examined the number of access points overall.

Furthermore, scholarship in this area has seldom disaggregated these connections by racial and ethnic groups. Studies that include race and ethnicity self-categorization as predictors of web-based activities [21] can unearth patterns of devices and contexts of use, such as Black (vs non-Black) individuals using mobile devices more often [24]. However, disparities among intersectional identities may still be overlooked, such as how age and race may interact to affect technology use [27]. Therefore, it is unclear where disparities in OHIS exist among intersectional identities.

**Figure 1:**
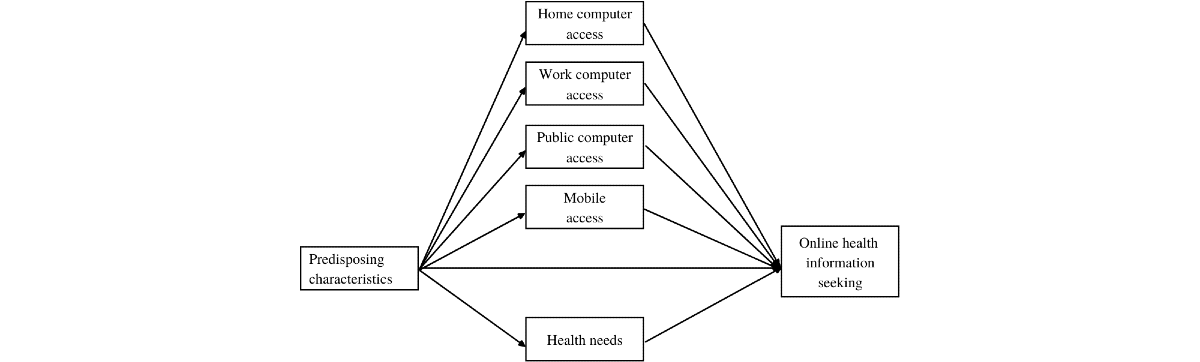
Initial online health information seeking model.

Previous OHIS theorizing [14,28] highlights several factors that influence health-seeking behaviors. As such, our model includes predisposing characteristics (age, sex, education, and income), access (home computer, work computer, public computer, and mobile), health needs, and OHIS (Figure 1). Our model also focuses on the foundational level of the digital divide (ie, access). As such, our nuanced conceptualization of access helps to fill the empirical gap in OHIS research on the first-level digital divide [29]. Thus, this study offers 2 primary contributions. First, we apply a multidimensional conceptualization of access along dimensions of the context of use (eg, at home vs in public) and device type (eg, smartphone vs computer) to theoretical models of OHIS. Second, we disaggregate our models by race and ethnicity to examine how they manifest differently across racial and ethnic groups. We explicate the relationships between predisposing characteristics, access, and health needs in our model.

### Antecedents of OHIS

#### Predisposing Characteristics

First, we posit associations between OHIS and predisposing characteristics. Younger individuals are more likely than older individuals to be able to navigate web-based platforms to seek out web-based health information [30,31]. Females are often expected to seek out health information because of their social roles as family caregivers [23,32]. Furthermore, education and income are generally positively associated with OHIS [33,34], as individuals with low education or income are inhibited from participating in OHIS because of low literacy [5]. Thus, we propose the following hypothesis:

Hypothesis 1 (H1): Demographic variables—age, sex, education, and income—will be associated with OHIS.

#### Internet Access

Expanding our understanding of OHIS, we extend prior conceptualizations of access [7,21,23] and examine access by considering context and device. *Context* refers to the physical environment in which users engage in OHIS, and *device* refers to the physical technology used to engage in OHIS. We focus on four of the most common context–device pairings: home computer, work computer, public computer, and mobile (which can be used across contexts). People seek health information on the web on several devices and at several places [23]. Owing to the extent that computers and mobile devices entail different technological constraints [8], and context structures media use [35], it is crucial to consider how different context–device pairings relate to OHIS.

First, *home computer* access involves computer use at home. It facilitates the availability of OHIS in a private setting where people spend most of their time [23]. However, because of the large cost of computers, ownership trends reflect the digital divide: White individuals are more likely to own home computers than Black and Latine individuals [3,7].

Second, *work computer* access involves computer use in the workplace. Individuals may not own these devices and likely would not pay for access, making it less expensive than home-computer access. However, work computer access requires employment that involves or entails access to computers [36]. The workplace is also a less frequented and private setting than the home [23,37].

Third, *public computer* access involves computer use in public facilities [3]. Such access can be inexpensive (if not free) and occurs in typically accessible public places, enabling access for individuals who cannot afford devices with internet access [21]. However, public computer access is contingent on a variety of factors, including the hours, locations, and resources of public facilities, which restrict the availability of such services [5].

Finally, *mobile* access involves the use of mobile devices (typically smartphones) in a variety of contexts. Mobile devices allow users to connect to public Wi-Fi and data networks, enabling OHIS in a variety of public, private, and (uniquely) mobile places (eg, on the bus) [38]. However, the small size of the mobile interface may restrict more intensive tasks [8], such as OHIS.

Overall, we expect that access will be related to predisposing characteristics and OHIS. Older individuals are less likely to access the internet [7,39], and males tend to access the internet more than females [18]. Education and income are positively correlated with access [40,41]. These differences may be linked to literacy and resources, allowing certain groups to maintain [7] and navigate access [10]. Furthermore, OHIS, by definition, is contingent on internet access [42,43]. With these considerations in mind, we propose the following hypotheses:

Hypothesis 2 (H2): Predisposing characteristics (ie, age, sex, education, and income) will be associated with access.

Hypothesis 3 (H3): Access will be positively associated with OHIS.

However, it is unclear how our nuanced conceptualization of access (ie, 4 discrete context–device pairings) may differentially affect OHIS. Thus, we pose the following research question (RQ):

RQ1: Which access pairings have the most consistent associations with OHIS across racial and ethnic groups?

#### Health Need

We conceptualize health needs as the extent to which individuals perceive that they require current or chronic medical attention. The likelihood that one may endure chronic illness is linked to group identities along the lines of age, gender, education level, and income [44]. When avoidable health differences in treatment, access to treatment, mortality, and diseases correlate with group identity, a health disparity occurs. Older individuals report greater health needs than younger individuals [45]. Although health disparities among males and females differ based on the illness, males may be more confident in their ability to maintain their health and report lower health needs [46]. Braveman at al [44] highlight that education level and income are important health determinants that predict health needs. Finally, individuals who perceive their health to be poor often demonstrate motivation to find health information on the web [28,33]. Thus, we propose the following hypotheses:

Hypothesis 4 (H4): Predisposing characteristics (ie, age, sex, education, and income) will be associated with health needs.

Hypothesis 5 (H5): Health needs will be positively associated with OHIS.

#### Race and Ethnicity

This study holds that existing racial and ethnic disparities exacerbate the impact of the digital divide on health disparities [22]. As such, we investigate how these inequities may affect OHIS. We explore whether hypotheses linking predisposing characteristics with OHIS (H1), access (H2), and health need (H4) differ across racial and ethnic groups. First, race and ethnicity may interact with age, sex, education, and income to predict OHIS as unique disparities in health and technology have been observed within groups that have intersecting predisposing characteristics and racial and ethnic group identities [41]. Next, race and ethnicity may interact with access to predict OHIS. Fang et al [47] provide illustrative insights, highlighting that age was a strong predictor for access but that this effect was exaggerated for some racial and ethnic minorities. Finally, race and ethnicity may interact with health needs to predict OHIS. For example, Black and Latine individuals are more likely to live in low-income areas [4,6], which is associated with exacerbated health needs [48]. As such, this study foregrounds the persistent racial and ethnic disparities in the United States to understand access and health needs from an intersectionality perspective [49].

In addition, race and ethnicity may interact with access (H3) and health need (H5) to influence OHIS. Regarding access, even when Black and Latine individuals access the internet at similar rates as White individuals, such access is often marked by greater insecurity [39]. Similarly, even with similar levels of health needs, racial and ethnic minorities may avoid seeking out web-based health information if they possess lower health and technology literacy [50,51]. Overall, our study uses previously tested models of OHIS [14,28,50-52] and seeks to extend previous theories by applying a multidimensional conceptualization of access and testing the fit of the model across racial and ethnic groups. These intersectional considerations, heightened by higher rates of internet insecurity [39] and greater health needs [4,6] because of systemic inequality, beg the following question:

RQ2: How will the relationships between predisposing characteristics, access, health needs, and OHIS differ across different racial and ethnic groups?

## Methods

### Sample

To test our model, we used data from the 2019 HINTS, also known as HINTS 5, Cycle 3 [11]. HINTS is an annual, nationally representative survey that asks participants about their engagement with health information. Data were collected between January 2019 and April 2019. A total of 5438 individuals responded to the survey. However, of the 5438 responses, 191 (3.51%) responses were deemed ineligible by HINTS because of partial completion, leaving 5247 (96.49%) individuals. Participants who did not complete the self-categorization variables for each model were excluded (Black and White: 420/5247, 8%; Latine: 487/5247, 9.28%). In addition, participants who did not complete all model variables were excluded (Black and White: 446/5247, 8.12%; Latine: 408/5247, 7.78%). Taken together, the sample size for the final models’ group was 4381 (based on Black and White self-categorization) or 4352 (based on Latine self-categorization). Owing to these different sample sizes, we report demographics and correlations for the 5247 individuals deemed eligible by HINTS. Only the variables presented in the *Measures* section were used for the purposes of this study; all other variables were excluded. Data are available in Multimedia Appendix 1. More information regarding the methodology can be found in the 2019 HINTS methodology report [53].

### Ethical Considerations

An institutional review board approval was not requested because the analysis for this study was conducted using secondary data. All HINTS data sets, including the one used for analysis in this study, have been approved through expedited review by the Westat Institutional Review Board, and subsequently deemed exempt by the U.S. National Institutes of Health Office of Human Subjects Research Protections [54].

### Participant Demographics

Demographic data were used to assess predisposing factors. Participants were aged 56.58 (SD 16.88) years on average. Approximately 56.62% (2971/5247) of the participants self-categorized as female, and 41.16% (2160/5247) self-categorized as male. Race and ethnicity were operationalized in comparison with those who did not self-categorize as the respective racial or ethnic group as individuals who self-categorize ethnically as Latine may still self-categorize racially as White or Black. Of the 5247 individual, 3727 (71.03%) self-categorized as White, and 1100 (20.96%) did not; 847 (16.14%) self-categorized as Black and 3980 (75.85%) did not; and 716 (13.64%) participants self-categorized as Latine and 4044 (77.07%) did not. The remaining individuals did not disclose their sex, race, or ethnicity. Participants’ level of education was measured on a 5-point scale from *less than high school* (score=1) to *postbaccalaureate degree* (score=5), and participants’ annual income was measured on a 7-point scale from *US* *$0* to *US $19,999* (score=1) to *≥ US $200,000* (score=7). See Table 1 for a summary of participant demographics.

**Table 1:** Participant demographics.

Demographics			OHIS^a,b^		
			No, n (%)		Yes, n (%)
**Age (years; n=5090)**					
	18-24	20 (0.39)		132 (2.59)	
	25-35	62 (1.22)		535 (10.51)	
	36-44	66 (1.30)		492 (9.67)	
	45-54	172 (3.38)		627 (12.32)	
	55-64	316 (6.21)		827 (16.25)	
	≥65	790 (15.52)		1051 (20.65)	
**Sex (n=5110)**					
	Male	652 (12.76)		1501 (29.37)	
	Female	798 (15.62)		2159 (42.25)	
**White (n=4805)**					
	No	344 (7.16)		746 (15.53)	
	Yes	977 (20.33)		2738 (56.98)	
**Black (n=4805)**					
	No	1034 (21.52)		2934 (61.06)	
	Yes	287 (5.97)		550 (11.45)	
**Latine (n=4745)**					
	No	1015 (21.39)		3016 (63.56)	
	Yes	237 (4.99)		477 (11.45)	
**Education** **(n=5087)**					
	Less than high school	200 (3.93)		108 (2.12)	
	High school graduate	445 (8.75)		448 (8.81)	
	Some college	441 (8.67)		1093 (21.49)	
	Received a bachelor’s degree	230 (4.52)		1130 (22.21)	
	Received a postbaccalaureate degree	117 (2.30)		875 (17.2)	
**Income (US $; n=4637)**					
	0-19,999	411 (8.86)		441 (9.51)	
	20,000-34,999	213 (4.59)		380 (8.19)	
	35,000-49,999	173 (3.73)		433 (9.34)	
	50,000-74,999	182 (3.92)		639 (13.78)	
	75,000-99,999	116 (2.5)		461 (9.94)	
	100,000-199,999	106 (2.29)		764 (16.48)	
	>200,000	36 (0.78)		282 (6.08)	

^a^OHIS: online health information seeking.

^b^Percentages reflect those who responded to the OHIS item.

### Measures

#### Overview

Correlations between all variables are displayed in Tables 2-4.

**Table 2:** Descriptive statistics and correlations between study variables (age, sex, and White).

Predictors	Values, mean (SD)	Age		Sex		White	
		*r*	*P* value	*r*	*P* value	*r*	*P* value
Age (years)	56.58 (16.88)	1	—^a^	—	—	—	—
Sex^b^	0.42 (0.49)	0.04	.01	1	—	—	—
White^c^	0.77 (0.42)	0.04	.005	0.07	<.001	1	—
Black^d^	0.18 (0.38)	–0.01	.64	–0.09	<.001	–0.79	<.001
Latine^e^	0.15 (0.36)	–0.10	<.001	0.01	.68	0.10	<.001
Education	3.36 (1.16)	–0.17	<.001	0.03	.07	0.08	<.001
Income	3.76 (1.93)	–0.17	<.001	0.12	<.001	0.14	<.001
Home computer	1.15 (0.84)	–0.17	<.001	0.10	<.001	0.15	<.001
Work computer	0.70 (0.90)	–0.45	<.001	0.03	.06	0.06	<.001
Public computer	0.16 (0.39)	–0.20	<.001	0.01	.69	–0.07	<.001
Mobile	1.27 (0.85)	–0.51	<.001	–0.04	.004	0.08	<.001
Health needs	2.58 (0.94)	0.16	<.001	–0.02	.29	–0.09	<.001
Online health information seeking (OHIS)	0.71 (0.45)	–0.31	<.001	–0.04	.01	0.05	.001

^a^Not applicable.

^b^Coded as female=0 and male=1.

^c^Coded as non-White=0 and White=1.

^d^Coded as non-Black=0 and Black=1.

^e^Coded as non-Latine=0 and Latine=1.

**Table 3:** Descriptive statistics and correlations between study variables (Black, Latine, education, and income).

Predictors	Black		Latine		Education		Income	
	*r*	*P* value	*r*	*P* value	*r*	*P* value	*r*	*P* value
Age (years)	—^a^	—	—	—	—	—	—	—
Sex^b^	—	—	—	—	—	—	—	—
White^c^	—	—	—	—	—	—	—	—
Black^d^	1	—	—	—	—	—	—	—
Latine^e^	–0.10	<.001	1	—	—	—	—	—
Education	–0.12	<.001	–0.17	<.001	1	—	—	—
Income	–0.20	<.001	–0.12	<.001	0.47	<.001	1	—
Home computer	–0.15	<.001	–0.16	<.001	0.41	<.001	0.38	<.001
Work computer	–0.09	<.001	–0.08	<.001	0.40	<.001	0.46	<.001
Public computer	0.07	<.001	–0.01	.76	0.11	<.001	–0.03	.03
Mobile	–0.08	<.001	–0.04	.005	0.33	<.001	0.37	<.001
Health need	0.10	<.001	0.07	<.001	–0.25	<.001	–0.31	<.001
Online health information seeking (OHIS)	–0.07	<.001	–0.07	<.001	0.34	<.001	0.28	<.001

^a^Not applicable.

^b^Coded as female=0 and male=1.

^c^Coded as non-White=0 and White=1.

^d^Coded as non-Black=0 and Black=1.

^e^Coded as non-Latine=0 and Latine=1.

**Table 4:** Descriptive statistics and correlations between study variables (home computer, work computer, public computer, mobile, and health need).

Predictors	Home computer			Work computer			Public computer			Mobile			Health need	
	*r*	*P* value	*r*		*P* value	*r*		*P* value	*r*		*P* value	*r*		*P* value
Age (years)	—^a^	—	—		—	—		—	—		—	—		—
Sex^b^	—	—	—		—	—		—	—		—	—		—
White^c^	—	—	—		—	—		—	—		—	—		—
Black^d^	—	—	—		—	—		—	—		—	—		—
Latine^e^	—	—	—		—	—		—	—		—	—		—
Education	—	—	—		—	—		—	—		—	—		—
Income	—	—	—		—	—		—	—		—	—		—
Home computer	1	—	—		—	—		—	—		—	—		—
Work computer	0.38	<.001	1		—	—		—	—		—	—		—
Public computer	0.15	<.001	0.13		<.001	1		—	—		—	—		—
Mobile	0.48	<.001	0.48		<.001	0.21		<.001	1		—	—		—
Health need	–0.18	<.001	–0.23		<.001	–0.02		.14	–0.20		<.001	1		—
Online health information seeking (OHIS)	0.41	<.001	0.30		<.001	0.14		<.001	0.46		<.001	–0.10		<.001

^a^Not applicable.

^b^Coded as female=0 and male=1.

^c^Coded as non-White=0 and White=1.

^d^Coded as non-Black=0 and Black=1.

^e^Coded as non-Latine=0 and Latine=1.

#### Internet Access

Participants reported how often they access the internet on a computer at home, at work, in a public place, and on a mobile device. A single item was used to measure each mode of access. Items were measured on 3-point scales, including *not applicable or never* (score=0), *sometimes* (score=1), and *daily* (score=2). There were varied responses for home computer (mean 1.14, SD 0.84), work computer (mean 0.70, SD 0.90), public computer (mean 0.16, SD 0.39), and mobile (mean 1.27, SD 0.85) access.

#### Health Need

Health needs were operationalized as perceived general health [14]. Thus, it was measured with a single item: “In general, how would you say your health is?” The item was measured on a 5-point scale from *excellent* (score=1) to *poor* (score=5; mean 2.58, SD 0.94). As measured, greater values represent greater health needs or poorer general health.

#### OHIS Measure

Participants reported using a single item, whether they used a computer, smartphone, or other electronic means to look for health or medical information for themselves in the past 12 months. Responses were *no* (score=0) or *yes* (score=1; mean 0.71, SD 0.45).

### Statistical Analysis

The initial demographic data were cleaned and analyzed using SPSS Statistics (version 27, IBM Corporation; Multimedia Appendix 1). Three group structural equation modeling models were specified based on the Black, Latine, and White self-categorization using Mplus 8.4 (Muthen and Muthén) [55]. Owing to the dichotomous outcome, diagonally weighted least squares mean and variance adjusted estimators were used instead of maximum likelihood to estimate the models, and odds ratios (ORs; vs standardized coefficients) were used to interpret relationships with OHIS. These models evaluated relationships between predisposing characteristics, access, health needs, and OHIS (H1-H5) and determined the access pairings most predictive of OHIS (RQ1). To test differences across racial and ethnic groups (RQ2), we constrained individual paths and compared each model with its respective baseline model, using chi-square tests for difference testing to account for the diagonally weighted least squares mean and variance estimation method.

## Results

### Model Fit

**Figure 2:**
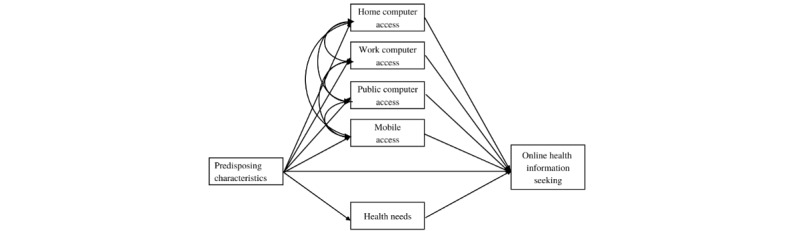
Final online health information seeking model.

Our proposed models grouped by Black, Latin, and White self-categorization displayed poor fit statistics [56]. Modification indices suggested the addition of correlations between all the access variables. Individuals who partake in OHIS are likely to do so in multiple ways [23,57]—thus, these correlations were incorporated into the models (Figure 2). The resulting models yielded appropriate fit statistics [56] for all 3 models, grouped by Black (root mean square error of approximation [RMSEA]=0.026; comparative fit index [CFI]=0.997; standardized root mean square residual [SRMR]=0.008), Latine (RMSEA=0.021; CFI=0.998; SRMR=0.007), and White (RMSEA=0.026; CFI=0.997; SRMR=0.007) self-categorization.

### Theoretical Model

First, we examined whether predisposing characteristics were associated with OHIS (H1). Age was negatively associated with OHIS, and education was positively associated with OHIS across all models and groups. Income was positively associated with OHIS for individuals who self-categorized as White (OR 1.122, 95% CI 1.048-1.202; *P*<.001), non-White (OR 1.121, 95% CI 0.989-1.271; *P*=.02), non-Black (OR 1.124, 95% CI 1.052-1.201; *P*<.001), and non-Latine (OR 1.123, 95% CI 1.051-1.200; *P*<.001). Sex was negatively associated with OHIS, such that White (OR 0.572, 95% CI 0.513-0.638; *P*<.001), non-Black (OR 0.591, 95% CI 0.530-0.660; *P*<.001), Latine (OR 0.601, 95% CI 0.474-0.762; *P*=.009), and non-Latine (OR 0.656, 95% CI 0.581-0.741; *P*<.001) females were more likely to engage in OHIS. These findings lent partial support for H1.

Next, we investigated whether predisposing characteristics were associated with access (H2) and whether access was associated with OHIS (H3). Age was negatively associated with all forms of access for all the models and groups. Education was positively associated with all forms of access for all models and groups, except public computer access for Latine individuals (*β*=.087; *P*=.10). Income was positively associated with home computer access, work computer access, and mobile access—but negatively associated with public computer access—for all models and groups. Sex was negatively associated with mobile access for all models and groups and negatively associated with work computer access for Black individuals (*β*=−.097; *P*=.003), such that females who self-categorized with these groups accessed the internet more frequently than males within these context–device pairings. However, sex was positively associated with home computer access for individuals who self-categorized as White (*β*=.071; *P*<.001), non-Black (*β*=.074; *P*<.001), and non-Latine (*β*=.063; *P*<.001) as well as public computer access for individuals who self-categorized as non-Black (*β*=.035; *P*=.04) and non-Latine (*β*=.033; *P*=.048), such that males who self-categorized with these groups accessed the internet more frequently than females. This provided partial support for H2.

Turning to OHIS, mobile and home access were positively associated with OHIS across all models and groups. Public computer access was positively associated with OHIS for non-White (OR 1.307, 95% CI 0.706-2.418; *P*=.04) individuals. Finally, work computer access was not associated with OHIS in any model and for any group. This provided partial support for H3. Regarding RQ1, mobile and home computer access were associated with OHIS more consistently across groups than public computer access and work computer access.

Then, we examined whether predisposing characteristics were associated with health needs (H4) and whether health needs were associated with OHIS (H5). Age was positively associated with health needs across all models and groups. Education and income were negatively associated with health needs across all models and groups, except for the relationship between education and health needs for non-White (*β*=−.061; *P*=.08) and Black (*β*=−.072; *P*=.06) individuals. Sex was not associated with health needs for any group. This provided partial support for H4. Turning to OHIS, health needs were positively associated with OHIS across all models and groups apart from non-White (OR 1.182, 95% CI 0.958-1.458; *P*=.07) and Latine (OR 1.091, 95% CI 0.869-1.369; *P*=.58) individuals. This provided partial support for H5.

### Intersectional Differences

Finally, we investigated whether significant differences in H1 to H5 emerged for different groups (RQ2). We found that certain relationships between predisposing characteristics and access differed for each type of access; all reported relationships were significant at *P*<.05. Significant differences emerged for the relationship between home computer access and age, income, and sex. The negative association with age was stronger for non-White, Black, and Latine individuals. The positive association with income was stronger for non-White and Black individuals. The association with sex was stronger for non-Black individuals; males were more likely to have home computer access than females among non-Black individuals. Significant differences also emerged for the association of work computer access with age and sex. The negative association with age was significantly stronger for non-Latine individuals. The association with sex was stronger for Black individuals; females were more likely to have work computer access than males among Black individuals. Next, the negative association between public computer access and age was stronger for non-White and Black individuals. Finally, the positive association between mobile access and education was significantly stronger for non-White and Latine individuals.

Other relationships also differed across racial and ethnic groups. For predisposing characteristics and OHIS, sex had a stronger negative association with OHIS for non-Black and White individuals, such that the gap between females and males engaging in OHIS was greater for these groups. For access and OHIS, home computer access had a significantly stronger positive association with OHIS for White, non-Black, and non-Latine individuals. Mobile access had a significantly stronger positive association with OHIS for non-White individuals. There were no significant differences in other dimensions of access or health needs. Tables 5-7 display the ORs and standardized coefficients for the final models, and our general analysis scripts are available in the Multimedia Appendix 1.

**Table 5:** Standardized coefficients and odds ratios for theorized OHIS^a^ models (for Black and non-Black individuals)^b^.

Path			Group				
			Black			Non-Black	
			Standardized coefficient^c^ or odds ratio^d^ (95% CI)	*P* value^e^	Standardized coefficient or odds ratio (95% CI)		*P* value
**Predisposing characteristics → OHIS**							
	Age (years)		0.982^f^ (0.967-0.996)	.003	0.976^f^ (0.970 -0.982)		<.001
	Sex		1.134^f^ (0.721-1.783)	.74	0.591^g^ (0.530-0.660)		<.001
	Income		1.081^f^ (0.939-1.245)	.15	1.124^f^ (1.052-1.201)		<.001
	Education		1.499^f^ (1.126-1.995)	<.001	1.439^f^ (1.259-1.644)		<.001
**Predisposing characteristics → access**							
	**Home computer**						
		Age (years)	–0.146^f^	<.001	–0.056^g^		<.001
		Sex	–0.036^f^	.44	0.074^g^		<.001
		Income	0.329^f^	<.001	0.170^g^		<.001
		Education	0.213^f^	<.001	0.292^f^		<.001
	**Work computer**						
		Age (years)	–0.294^f^	<.001	–0.368^f^		<.001
		Sex	–0.097^f^	.003	0.018^g^		.20
		Income	0.374^f^	<.001	0.283^f^		<.001
		Education	0.177^f^	<.001	0.186^f^		<.001
	**Public computer**						
		Age (years)	–0.255^f^	<.001	–0.180^g^		<.001
		Sex	0.033^f^	.39	0.035^f^		.04
		Income	–0.122^f^	.003	–0.115^f^		<.001
		Education	0.104^f^	.01	0.135^f^		<.001
	**Mobile**						
		Age (years)	–0.446^f^	<.001	–0.444^f^		<.001
		Sex	–0.105^f^	<.001	–0.060^f^		<.001
		Income	0.226^f^	<.001	0.220^f^		<.001
		Education	0.167^f^	<.001	0.131^f^		<.001
**Access → OHIS**							
	Home computer		1.491^f^ (0.990-2.246)	.001	1.981^g^ (1.554-2.526)		<.001
	Work computer		0.877^f^ (0.663-1.161)	.55	0.939^f^ (0.822-1.073)		.92
	Public computer		1.368^f^ (0.692-2.706)	.06	1.118^f^ (0.801-1.560)		.16
	Mobile		2.158^f^ (1.175-3.962)	<.001	1.833^f^ (1.440-2.333)		<.001
**Predisposing Characteristics → health need**							
	Age (years)		0.098^f^	.005	0.102^f^		<.001
	Sex		0.001^f^	.98	0.022^f^		.16
	Income		–0.202^f^	<.001	–0.245^f^		<.001
	Education		–0.072^f^	.06	–0.117^f^		<.001
**Health need → OHIS**			1.235^f^ (0.970-1.572)	.03	1.222^f^ (1.078-1.385)		.004

^a^OHIS: online health information seeking.

^b^Comparisons were made for each model per row.

^c^Standardized coefficients are displayed for paths predicting nondichotomous outcomes; negative relationships are indicated by negative signs. For standardized coefficients, 95% CI values are not available.

^d^Odds ratios are presented for paths predicting dichotomous outcomes (ie, OHIS) and were generated using Monte Carlo integration because of model complexity; negative relationships are indicated by values <1.

^e^Significance values were based on the primary models (ie, without Monte Carlo integration).

^f^Coefficients or odds ratios differ significantly from those denoted by footnote *g* at *P*<.05.

^g^Coefficients or odds ratios differ significantly from those denoted by footnote *f* at *P*<.05.

**Table 6:** Standardized coefficients and odds ratios for theorized OHIS^a^ models (for Latine and non-Latine individuals)^b^.

Path				Group						
				Latine				Non-Latine		
				Standardized coefficient^c^ or odds ratio^d^ (95% CI)		*P* value^e^		Standardized coefficient or odds ratio (95% CI)		*P* value
**Predisposing characteristics → OHIS**										
	Age (years)		0.979^f^ (0.966-0.993)		.002		0.977^f^ (0.971-0.983)		<.001	
	Sex		0.601^f^ (0.474-0.762)		.009		0.656^f^ (0.581-0.741)		<.001	
	Income		1.058^f^ (0.930-1.204)		.42		1.123^f^ (1.051-1.200)		<.001	
	Education		1.393^f^ (1.075-1.804)		<.001		1.481^f^ (1.291-1.699)		<.001	
**Predisposing characteristics → access**										
	**Home computer**									
		Age (years)	–0.185^f^		<.001		–0.054^g^		<.001	
		Sex	0.060^f^		.08		0.063^f^		<.001	
		Income	0.181^f^		<.001		0.203^f^		<.001	
		Education	0.324^f^		<.001		0.254^f^		<.001	
	**Work computer**									
		Age (years)	–0.249^f^		<.001		–0.374^g^		<.001	
		Sex	0.019^f^		.57		–0.006^f^		.68	
		Income	0.265^f^		<.001		0.301^f^		<.001	
		Education	0.259^f^		<.001		0.171^f^		<.001	
	**Public computer**									
		Age (years)	–0.257^f^		<.001		–0.188^f^		<.001	
		Sex	–0.024^f^		.57		0.033^f^		.048	
		Income	–0.152^f^		.002		–0.142^f^		<.001	
		Education	0.087^f^		.10		0.128^f^		<.001	
	**Mobile**									
		Age (years)	–0.462^f^		<.001		–0.439^f^		<.001	
		Sex	–0.069^f^		.03		–0.072^f^		<.001	
		Income	0.139^f^		<.001		0.232^f^		<.001	
		Education	0.206^f^		<.001		0.114^g^		<.001	
**Access → OHIS**										
	Home computer		1.294^f^ (0.861-1.945)		.04		1.950^g^ (1.541-2.467)		<.001	
	Work computer		1.121^f^ (0.772-1.627)		.19		0.917^f^ (0.806-1.044)		.49	
	Public computer		1.500^f^ (0.574-3.919)		.08		1.117^f^ (0.824-1.513)		.15	
	Mobile		1.739^f^ (1.057-2.861)		<.001		1.912^f^ (1.491-2.452)		<.001	
**Predisposing characteristics → health need**										
	Age (years)		0.140^f^		<.001		0.093^f^		<.001	
	Sex		–0.039^f^		.30		0.028^f^		.07	
	Income		–0.178^f^		<.001		–0.243^f^		<.001	
	Education		–0.147^f^		<.001		–0.113^f^		<.001	
**Health need → OHIS**				1.091^f^ (0.869-1.369)		.58		1.242^f^ (1.093-1.411)		.001

^a^OHIS: online health information seeking.

^b^Comparisons were made for each model per row.

^c^Standardized coefficients are displayed for paths predicting nondichotomous outcomes; negative relationships are indicated by negative signs. For standardized coefficients, 95% CI values are not available.

^d^Odds ratios are presented for paths predicting dichotomous outcomes (ie, OHIS) and were generated using Monte Carlo integration because of model complexity; negative relationships are indicated by values <1.

^e^Significance values were based on the primary models (ie, without Monte Carlo integration).

^f^Coefficients or odds ratios differ significantly from those denoted by footnote *g* at *P*<.05.

^g^Coefficients or odds ratios differ significantly from those denoted by footnote *f* at *P*<.05.

**Table 7:** Standardized coefficients and odds ratios for theorized OHIS^a^ models (for White and non-White individuals)^b^.

Path			Group				
			White			Non-White	
			Standardized coefficient^c^ or odds ratio^d^ (95% CI)	*P* value^e^	Standardized coefficient or odds ratio (95% CI)		*P* value
**Predisposing characteristics → OHIS**							
	Age (years)		0.975^f^ (0.967-0.983)	<.001	0.982^f^ (0.971-0.994)		<.001
	Sex		0.572^f^ (0.513-0.638)	<.001	1.123^g^ (0.757-1.665)		.84
	Income		1.122^f^ (1.048-1.202)	<.001	1.121^f^ (0.989-1.271)		.02
	Education		1.456^f^ (1.267-1.673)	<.001	1.427^f^ (1.119-1.819)		<.001
**Predisposing characteristics → access**							
	**Home computer**						
		Age (years)	–0.047^f^	.003	–0.158^g^		<.001
		Sex	0.071^f^	<.001	0.014^f^		.62
		Income	0.176^f^	<.001	0.265^g^		<.001
		Education	0.282^f^	<.001	0.280^f^		<.001
	**Work computer**						
		Age (years)	–0.370^f^	<.001	–0.298^f^		<.001
		Sex	–0.001^f^	.95	0.010^f^		.71
		Income	0.289^f^	<.001	0.329^f^		<.001
		Education	0.182^f^	<.001	0.205^f^		<.001
	**Public computer**						
		Age (years)	–0.178^f^	<.001	–0.242^g^		<.001
		Sex	0.033^f^	.06	0.038^f^		.26
		Income	–0.122^f^	<.001	–0.129^f^		<.001
		Education	0.131^f^	<.001	0.126^f^		.001
	**Mobile**						
		Age (years)	–0.450^f^	<.001	–0.432^f^		<.001
		Sex	–0.069^f^	<.001	–0.065^f^		.01
		Income	0.223^f^	<.001	0.202^f^		<.001
		Education	0.121^f^	<.001	0.199^g^		<.001
**Access → OHIS**							
	Home computer		2.002^f^ (1.558-2.573)	<.001	1.458^g^ (1.005-2.116)		<.001
	Work computer		0.921^f^ (0.806-1.052)	.63	0.931^f^ (0.712-1.218)		.96
	Public computer		1.098^f^ (0.787-1.532)	.25	1.307^f^ (0.706-2.418)		.037
	Mobile		1.776^f^ (1.398-2.256)	<.001	2.379^g^ (1.281-4.419)		<.001
**Predisposing characteristics → health need**							
	Age (years)		0.097^f^	<.001	0.123^f^		<.001
	Sex		0.027^f^	.09	–0.015^f^		.64
	Income		–0.246^f^	<.001	–0.226^f^		<.001
	Education		–0.124^f^	<.001	–0.061^f^		.08
**Health need → OHIS**			1.237^f^ (1.085-1.411)	.002	1.182^f^ (0.958-1.458)		.07

^a^OHIS: online health information seeking.

^b^Comparisons were made for each model per row.

^c^Standardized coefficients are displayed for paths predicting nondichotomous outcomes; negative relationships are indicated by negative signs. For standardized coefficients, 95% CI values are not available.

^d^Odds ratios are presented for paths predicting dichotomous outcomes (ie, OHIS) and were generated using Monte Carlo integration because of model complexity; negative relationships are indicated by values <1.

^e^Significance values were based on the primary models (ie, without Monte Carlo integration).

^f^Coefficients or odds ratios differ significantly from those denoted by footnote *g* at *P*<.05.

^g^Coefficients or odds ratios differ significantly from those denoted by footnote *f* at *P*<.05.

## Discussion

### Principal Findings

This study applied a nuanced conceptualization of access to theoretical models of OHIS and identified how relationships with OHIS differed between racial and ethnic groups (ie, Black, Latine, and White individuals). We found partial support for all hypotheses, and results regarding the RQs provided deeper insight into the predicted relationships. By examining access as 4 unique context–device pairings, we found that home computer and mobile access were most consistently associated with OHIS. In addition, disaggregating models by racial and ethnic self-categorization identified different patterns between predisposing characteristics and access for different groups, highlighting how the digital divide affects intersectional groups.

Our findings suggest that predisposing characteristics are associated with OHIS for different racial and ethnic groups (H1). Education was positively associated with OHIS, and age was negatively associated with OHIS. These findings align with previous research, such that those with more education and younger individuals are more likely to possess the skills to navigate web-based platforms [30,31,33,58]. However, income only had a positive association with OHIS for individuals who self-categorized as White, non-Black, or non-Latine. Although education and income are often correlated with OHIS [9,59,60], our findings suggest that education may better index the foundational knowledge necessary to take advantage of web-based health information. Finally, although females sought out health information more frequently than males [23,32], this pattern did not hold for those who self-categorized as Black. This may reflect how factors of socioeconomic status are typically stronger determinants of access to technology and health services for racial minorities than sociodemographic factors [44,61].

Our findings also suggest that some predisposing characteristics are associated with access for some racial and ethnic groups (H2). Age was negatively associated with all forms of access. Older individuals used all 4 context–device pairings less frequently than younger individuals, which may indicate their use of nondigital means (eg, print media and interpersonal) to obtain health information [47]. Females of all groups accessed the internet on mobile devices more frequently than males, as well as work computers among Black individuals. However, White males (vs females) accessed the internet on home computers more frequently. Although males and females may have similar access overall [62], combining a multifaceted conceptualization of access with an intersectional approach highlights that disparities in access based on sex are grounded in devices and contexts of use, as well as race and ethnicity. White males and females are more likely to access the internet on home computers and mobile devices, respectively, suggesting that internet access may be a zero-sum game, such that having access in one place reduces the need to have access elsewhere [21]. However, this trade-off did not emerge for other groups, suggesting important boundary conditions based on race and ethnicity [61]. Furthermore, income and education consistently demonstrated a positive association with access, as maintaining access requires sustainable resources afforded by income and education [7]. However, income was negatively associated with public computer access, suggesting that individuals with lower income may be more reliant on public resources to access the internet [39,57].

Our findings generally confirm that access is associated with OHIS (H3). As suggested by previous research [14,28,50-52], OHIS is unlikely without a means to access the internet. Specifically, mobile access was positively associated with OHIS for all groups, suggesting that the ubiquity of mobile phones may help bridge this particular gap of the digital divide [3,5,21]. Home computer access was also associated with OHIS for all groups. Public computer access was positively associated with OHIS for non-White individuals. Work computer access was not associated with OHIS across all groups. These findings are corroborated by the fact that certain contexts of OHIS (eg, home computer and mobile) provide a level of privacy that other contexts do not [23,37], thus facilitating searches for private health information. Our results highlight that certain groups (particularly non-White individuals) face access disparities based on affordance and maintenance of that privacy [7,63].

Predisposing characteristics were also associated with health needs (H4), such that older individuals and individuals with less education and income were more likely to describe their health as poor. Older individuals and individuals with less education and income often face barriers to quality health options [44]. However, education was not significantly associated with health needs for Black and non-White individuals. Research on minority groups (eg, Black individuals) finds that educational advancement may not overcome the aggregated stress of marginalization, which contributes to negative health outcomes [64]. However, sex was not associated with health needs for any group, likely because of counterbalancing of health issues that disproportionately affect males and females separately [45].

Furthermore, those with greater health needs were more likely to partake in OHIS, apart from non-White and Latine individuals (H5). Past research has found that greater health needs are associated with increased OHIS among Latine individuals [58]. However, our findings support previous findings that Latine individuals may be less trusting of health information on the web and may rely on different (eg, interpersonal) means of seeking out health information [65]. Reconciliation of these contradicting findings may be a result of area, as the national sample is not limited to patterns that may only exist in larger cities with more resources to provide access [58].

Finally, our exploratory analyses provide insight into RQ2; however, additional research may be required to fully explicate certain patterns in our model in which stronger relationships were detected for specific racial and ethnic groups. In terms of access, several relationships were stronger for Black individuals. Greater income was associated with more frequent home computer access across all groups; however, this relationship was stronger for Black (vs non-Black) individuals. Income inequality among Black individuals appears to be a stark determinant of home computer access [39]. Individuals with higher income can afford the cost of maintenance that comes with home computer access, which is apparent across the models [7]. However, Black individuals with lower income may face additional hurdles to home computer access, such as living in areas without the infrastructure to support maintenance [3]. Similarly, the negative relationship between age and public computer access was stronger for Black (vs non-Black) individuals. The restricted availability of public computers [5] and limited accessibility of web-based platforms for older individuals [7] may be particularly profound for Black individuals. As Black individuals have been historically disadvantaged in access, both community resources and technological skills of the older generation may be stunted [5,39]. Furthermore, the relationship between sex and access differed, such that Black females reported more frequent access via work computers than Black males. In contrast, non-Black males were more likely to use home computers. For specific sex, racial, and ethnic groups, finding access to the internet via 1 mode may be sufficient, which could reduce the need to have access elsewhere [21]. Finally, non-White (vs White) individuals, or racial and ethnic minorities in general, demonstrated a stronger negative relationship between age and home computer access and a stronger positive relationship between education and mobile access. Older racial and ethnic minorities tend to have less access to the internet [39], including at home. Although racial and ethnic minorities lag in home computer ownership, the stronger relationship with education and mobile access may be interpreted as a route to attenuate the digital divide or as exacerbating the digital divide within racial minority groups. As such, lower education levels seem to inhibit access more intensely among Latine individuals.

In addition to access, the relationship between sex and OHIS differed, such that non-Black (vs Black) females demonstrated a stronger association with OHIS. The extent to which females relieve the burden of family health knowledge [32] may differ across racial and ethnic groups, as these groups are often disproportionately affected by health disparities [4,6]. Moreover, the positive relationship between home computer access and OHIS was weaker for non-White (vs White) individuals, whereas the positive relationship between mobile access and OHIS was stronger. Mobile devices remained a key factor not only for establishing access for racial and ethnic minorities [3] but also for OHIS. Although the mobile interface may be more difficult for tasks such as OHIS [8], having at least one point of access is critical for web-based health behaviors [9,24]. Although some OHIS research suggests that access has been democratized [19], the above results highlight the overlap in health and technology disparities for racial and ethnic minority groups [4-6].

### Contributions

Our first contribution—applying a multidimensional conceptualization of access to theoretical models of OHIS—revealed that different context–device pairings offer distinct OHIS profiles. Mobile and home computer access were more consistently associated with OHIS than work computer and public computer access. This implies that privacy is important when assessing the digital divide, as home computers and mobile devices can be used in more private contexts [23,37]. Furthermore, for racial and ethnic minority participants, the link between home computer access and OHIS was weaker, and the relationship between mobile access and OHIS was stronger. These differences primarily emerged in the non-White versus White models because of reduced power in the other models and the fact that Black individuals were included as non-Latine individuals (and vice versa). Racial and ethnic minorities may rely more on mobile (vs home computer) access for OHIS because of the lower cost and flexibility of mobile devices [66]. Public and work computer access were not consistently associated with OHIS, although public computer access was generally associated with OHIS for non-White individuals. Contexts that typically do not require ownership may provide access for groups that lack other means of access. Discrepancies among home computer, work computer, public computer, and mobile access highlight that devices and contexts of use do not provide access equally [7].

Our second contribution was to unpack the digital divide using an intersectional approach, as it is crucial to understand which groups have limited access to the internet. We found discrepancies in access for specific groups. Older individuals who self-categorized as a racial or ethnic minority engaged in less frequent home and public computer access. Older (vs younger) individuals and racial and ethnic minorities (vs majorities) tend to access the internet less frequently [39], and we find that this gap is magnified for home and public computer access. In addition, for non-White (vs White) individuals, there were stronger positive relationships between education and mobile access, as well as mobile access and OHIS. Although formal education may minimize the digital divide via mobile access, disparities in access to the education needed to operate the devices should also be considered. Discrepancies between predisposing characteristics, access, health needs, and OHIS for different racial and ethnic groups demonstrate the need for future OHIS theorizing to adopt an intersectional approach.

### Practical Implications

This study supports the criticism that the digital divide is not a dichotomy between access and lack thereof [22]. In response to this criticism, interventions combating the digital divide must consider how context–device access pairings can be leveraged among specific racial and ethnic groups. Home computer and mobile were the most frequently used means of access and were both consistently positively related to OHIS. This implies that people typically engage in OHIS on home computers or mobile devices. As such, improving access within these contexts may be valuable for interventions to support OHIS across racial and ethnic groups. Our findings suggest that work and public computer access are less ideal for OHIS. These pairings may lack accessibility [5] or privacy [23,37]. The association between public computer access and OHIS for racial and ethnic minorities could be explored further as a means of increasing OHIS for Latine and Black individuals. Work and public computer access remain important to the extent that increasing access points overall supports OHIS [21,25,26]. However, public computer access may also replace more expensive yet more private modes of access (eg, home computer). In general, interventions could reinforce existing strengths (eg, home computer and mobile access) or bolster existing weaknesses (eg, work and public computer access).

These 2 courses of action can also apply to future interventions aimed at addressing the digital divide and OHIS among specific groups. For racial and ethnic minorities, we found weaker positive relationships between home computer access and OHIS and stronger positive relationships between mobile access and OHIS. Interventions can strengthen the established relationship between mobile devices and OHIS or bolster the weaker link for home computer access. Although home computer access is considered a more easily navigable interface [8], it may not be scalable, given its cost. As such, interventions with limited financial resources may benefit from working with providers of web-based health information to develop mobile-friendly interfaces to make health information more accessible. Furthermore, this study shines a spotlight on older racial and ethnic minorities, who experienced consistent discrepancies in access and may have the highest health needs. The negative relationship between age and public computer access was stronger for Black individuals, suggesting a drop-off in public computer access for older Black adults. As public computer access was associated with OHIS for racial and ethnic minorities, future interventions could increase the accessibility of public computers for older Black adults, with attention toward local libraries and community centers in predominately older Black neighborhoods [5]. Overall, a deep understanding of current community strengths must be balanced with efforts to provide equitable access to web-based health information to not overlook the key interactions between multiple social positions that create compounding experiences of oppression [67].

### Limitations and Future Research

Some limitations should be considered when interpreting these findings. This study used secondary cross-sectional data. Thus, potentially relevant variables (eg, mobile use at home vs at work vs in public) were not measured, and causality or directionality cannot be determined. Future research could measure additional constructs and use longitudinal designs. In addition, this analysis used self-reported data. Future work could use log and GPS data in tandem to paint a more accurate picture of OHIS. Furthermore, our primary outcome variable (OHIS) was dichotomous, and other variables (eg, access) were trichotomous or single-item measures. Future research should use continuous variables for OHIS and access to better capture the temporal variety of digital media use. Finally, we did not examine second-level digital divide variables (eg, experience, perceived utility, beliefs, and skills) [28,50,51,68,69]. However, before receiving and interpreting information, those who seek information on the web must choose a device and context to seek that information.

### Conclusions

This study holds that a nuanced conceptualization of access is necessary to understand how the digital divide differentially affects racial and ethnic groups. Our theoretical model identified variables that predict OHIS while distinguishing the type (ie, device) and location (ie, context) of access, testing these associations for different racial and ethnic groups and examining intersectional characteristics among these groups (ie, age, sex, education, and income). By interlacing a thorough understanding of the first-level digital divide with an awareness of the unique impacts of the digital divide for specific groups, we further theorize on OHIS and suggest important considerations for more targeted interventions. As we continue to understand the complexities of the digital divide and its relationship with health, racial, and ethnic disparities, our perspective highlights how web-based health resources may not be accessed by those who need them the most.

## Appendix

Multimedia Appendix 1 Data and syntax.

## Abbreviations

CFI: comparative fit index
HINTS: Health Information National Trends Survey
OHIS: online health information seeking
OR: odds ratio
RMSEA: root mean square error of approximation
RQ: research question
SRMR: standardized root mean square residual
